# The use of patient-reported outcome measures to improve patient-related outcomes – a systematic review

**DOI:** 10.1186/s12955-024-02312-4

**Published:** 2024-11-26

**Authors:** Joshua M. Bonsel, Ademola J. Itiola, Anouk S. Huberts, Gouke J. Bonsel, Hannah Penton

**Affiliations:** 1 Department of Orthopaedics and Sports Medicine, Erasmus Medical Center, University Medical Center Rotterdam, Doctor Molewaterplein 40, 3015 GD Rotterdam, The Netherlands; 2 School of Public Health, University of Alberta, Edmonton, AB Canada; 3 Department of Quality and Patientcare, Erasmus Medical Center, University Medical Center Rotterdam, Rotterdam, The Netherlands; 4 EuroQol Research Foundation, Rotterdam, The Netherlands; 5 OPEN Health Evidence & Access, Rotterdam, The Netherlands

**Keywords:** Patient-Reported Outcome Measures, Quality of care, Patient outcomes, Feedback, Monitoring, Benchmarking, Routine outcome measurement

## Abstract

**Background:**

Patient-reported outcome measures (PROMs) provide invaluable information on patients’ health outcomes and can be used to improve patient-related outcomes at the individual, organizational and policy levels. This systematic review aimed to a) identify contemporary applications and synthesize all evidence on the use of PROMs in these contexts and b) to determine characteristics of interventions associated with increased effectiveness.

**Methods:**

Five databases were searched for studies providing quantitative evidence of the impact of PROM interventions. Any study design was permitted. An overall benefit (worsening) in outcome was defined as a statistically significant improvement (deterioration) in either a PROM, patient-reported experience measure or clinical outcome. Study quality was assessed using the Effective Public Healthcare Panacea Project’s Quality Assessment Tool for Quantitative Studies. A narrative synthesis was conducted.

**Results:**

Seventy-six studies of the 11,121 articles identified met the inclusion criteria. At the individual level, 10 (43%) of 23 studies that fed back PROMs to the patient or healthcare provider showed an improvement in outcome. This percentage increased in studies which used PROMs to monitor disease symptoms and linked these to care-pathways: 17 (68%) of 25 studies using this mechanism showed an improvement. Ten (71%) of 14 studies using PROMs to screen for disease found a benefit. The monitoring and screening approach was most effective using PROMs covering cancer-related, depression and gastro-intestinal symptoms. Three studies found that the mere collection of PROMs resulted in improved outcomes. Another three studies used PROMs in decision aids and found improved decision quality.

At the organizational/policy level, none of the 4 studies that used PROMs for benchmarking found a benefit. The three studies that used PROMs for in-depth performance analyses and 1 study in a plan-do-study-act (PDCA) cycle found an improvement in outcome.

Studies employing disease-specific PROMs tended to observe improved outcomes more often. There are concerns regarding the validity of findings, as studies varied from weak to moderate quality.

**Conclusions:**

The use of PROMs at the individual level has matured considerably. Monitoring/screening applications seem promising particularly for diseases for which treatment algorithms rely on the experienced symptom burden by patients. Organizational/policy-level application is in its infancy, and performance evaluation via in-depth analyses and PDCA-cycles may be useful. The findings of this review may aid stakeholders in the development and implementation of PROM-interventions which truly impact patient outcomes.

**Supplementary Information:**

The online version contains supplementary material available at 10.1186/s12955-024-02312-4.

## Background

Patient-reported outcomes measures (PROMs) are considered an invaluable tool to capture information on patients’ health outcomes, including expectations and values. Two types of PROMs exist, namely generic and disease-specific PROMs [1]. Generic PROMs aim to measure a health outcome from an overarching perspective, allowing for comparison between different diseases and a general judgement on the severity. These measures are often multi-dimensional; examples include measures of overall Quality of Life (e.g., EQ-5D) or well-being (e.g., WHO-5) [2, 3]. Disease-specific PROMs aim to measure these concepts, the symptom burden and functional status associated with a disease or a group of diseases [4].

PROMs were introduced to complement clinical outcome measures in studies assessing the (cost-)effectiveness of new clinical interventions. However, their application has broadened, including the role as outcome indicator in clinical practice alongside traditional indicators such as mortality and prevalence/incidence [5]. This movement is adopted by medical science and leading institutions like the Organisation for Economic Co-operation and Development, which conform to the principle that assessing health system performance starts by assessment of patient-related outcomes [6]. It is pragmatic to distinguish three levels of intended use: the individual (micro-), organizational (meso-) and policy (macro-) level [7].

At the micro-level, PROMs are used at the patient-encounter level. Several systematic reviews revealed evidence that using PROMs at the micro-level has a modest beneficial impact on patient-related outcomes [8–15]. The key idea is that a patient fills out a PROM once or multiple times, and the results are fed back to the patient or clinician [15]. Greenhalgh et al. has outlined the underlying theory how PROMs may be useful at this level: the feedback of PROMs may alter the decision-making process, and initiate a change to clinical practice [16]. Several examples exist: firstly, the feedback of PROMs to patient and provider can aid in communicating symptoms which may otherwise remain unnoticed [17, 18]. Another example are novel digital patient-decision systems using PROMs, which develop rapidly parallel to digital technology (e.g., apps, e-portals, and dashboards) [19].

Aggregated PROMs can be used to inform the healthcare system at the organizational (meso-) and health system (macro-) level, respectively. Evidence of the impact of PROMs use at the meso-/macro-level is scarce, and a recent review did not find a clear impact on patient outcomes [8, 20]. The key idea at this level is that aggregated PROMs can guide the (continuous) improvement of healthcare provided by a group of clinicians, hospital or even country [21]. Their role in orthopedic surgery may illustrate their potential. At the meso-level, an orthopedic surgery unit in a hospital may use PROMs to improve local policy on eligibility criteria for surgical treatment, to rationalize pain killing strategies, or to compare performance across surgeons on a monthly basis [22]. At the macro-level, PROMs results according to hospital, region, nation, or otherwise may be presented in a standardized form (both in epidemiological and graphical meaning), inviting for a process of feedback, analysis of drivers, and if possible subsequent improvement [21]. This mechanism is often referred to as benchmarking and is thought to demonstrate performance differences among providers, facilitate more in-depth clinical audits, and inform decision-making, and is a potentially effective method to improve the quality of care [23, 24]. An example which aimed to encourage benchmarking is the NHS-programme in the UK on certain surgical procedures. This program publicly published PROMs for varicose vein, groin hernia, and hip and knee arthroplasty surgery; as of 2017 PROMs are only collected for hip/knee surgery [25]. This program also aimed to incentivize patients to select the assumed best provider, however, available evidence does not support this pathway [21, 26].

We think a contemporary review is warranted because it remains unknown why certain PROMs-interventions are more effective than others [8, 11]. Certain mechanisms underpinning the interventions may contribute to increased effectiveness. For example, a critical step to transform a suboptimal PROM level, i.e. a patient value below a particular threshold, into an improved outcome may be to link this observation to a care pathway. The doctor may receive an alert inviting her/him to check the situation. This approach seems promising in disease areas where symptom monitoring along with treatment tailoring is common practice, e.g., gastroenterology, rheumatology, and oncology [27, 28].

In this systematic review, we aim to identify contemporary evidence of the impact of the use of PROMs at the micro-, meso- and macro-level on patient outcomes. Our second aim is to identify and describe characteristics of the intervention and PROMs used which may contribute to an increased chance for success.

## Methods

The present systematic review was registered in PROSPERO under record 2022 CRD42022333400. This review followed the Preferred Reporting Items for Systematic Reviews and Meta-Analyses (PRISMA) guidelines (2020) when applicable [29].

### Data sources and search strategy

The following databases were searched: MEDLINE, Embase, Web of Science Core Collection, Cochrane CENTRAL Register of trials, and Google Scholar from database inception to August 24, 2023 for studies that reported the use of PROMs to improve quality of care. The final search was developed and refined through an iterative process and consisted of 3 blocks, namely: (a) various terms for PROMs, (b) various terms for quality, effectiveness and outcomes, and (c) mechanisms through which PROMs may be used to benefit healthcare (e.g., feedback, monitoring, dashboards and plan-do-check-act (PDCA) cycles) (Supplementary Material 1). A PDCA-cycle is a commonly used framework to guide the continuous improvement of healthcare and services provided [30]. Additional studies were identified by screening the references of included articles.

### Study selection

Studies were eligible that (a) provided evidence on the impact of an intervention, (b) using a previously validated PROM, (c) which reported at least one quantitative outcome per the definition described below. Any study design was permitted. Studies were excluded if (a) the full-text could not be retrieved and/or only a conference abstract was available; (b) the study was conducted as a pilot; (c) there was no comparator or pre-intervention comparison; (d) the PROM was used to select patients for another type of intervention; (e) the article was not available in English. Two reviewers (JB and AI) independently screened all titles and abstracts obtained from the search and applied the inclusion criteria to eligible studies. Any disagreements regarding the inclusion of studies was discussed between the two reviewers and were resolved by consensus.

### Outcome definition

We defined the potential impact of a PROM-intervention on patient-related outcomes using the Donabedian framework [31]. To evaluate the quality of healthcare or impact of an intervention, contemporary guidelines place emphasis on outcome measures which reflect the impact on the health status of patients [32]. Typically, these outcomes are of quantitative nature and are collected at the patient-level. We discerned three types of outcomes measured based on previous reviews, namely (1) PROMs, (2) patient reported experiences measures (PREMs) and (3) clinical outcomes. Outcome measures were categorized according to the dimensions/items into overarching groups based on the identified studies, e.g., Health-Related Quality of Life (HRQoL), physical functioning, mental functioning, and symptom burden. Similarly, this was done for PREMs (e.g., satisfaction) and clinical outcomes (e.g., readmissions).

A study was judged to have found an overall benefit (or a detriment/harm) if any of the above-mentioned outcomes improved (worsened) up to statistical significance. As patient-related outcomes may be specific to the intended use and medical domain, we did not attribute weight to a specific type of outcome. Studies often contained multiple comparisons through analysis of dimensions or even items separately. This approach inflates testing, increasing the potential of a type I error. Therefore, we required at least 2 subdomain/single-items to reach statistical significance to qualify the impact as a benefit or detriment, unless outcomes were defined as primary outcome a priori.

In accordance with previous reviews, process of care measures (e.g., number of symptoms discussed) were extracted, but were considered to mediate outcomes described above [14].

### Data extraction and quality assessment

The following data were extracted from eligible studies by one of the reviewers (JB or AI): authors, country, setting, study design, sample, PROMs used, description of intervention using PROMs, co-interventions, training offered on the intervention and/or interpretation of PROM, all primary and secondary outcome measures and their quantification.

Two reviewers (JB and AH) independently assessed the methodological quality of included studies using the Effective Public Healthcare Panacea Project’s Quality Assessment Tool for Quantitative Studies [33]. The tool was considered the most appropriate for this systematic review as it covers various study designs and public health interventions. Domains assessed using the tool included selection bias, study design, confounders, blinding, data collection methods, and withdrawals and drop-outs. Each domain was rated as 1 (strong), 2 (moderate) or 3 (weak). A global score was calculated, in which strong = no weak ratings, moderate = 1 weak rating, and weak = two or more weak ratings.

### Data synthesis

A narrative synthesis was conducted as a formal meta-analysis appeared not possible at an early stage due to the heterogeneity of study designs and outcomes reported. Overall, the synthesis was split up by the micro- and meso-/macro-level. The impact of PROMs interventions was assessed by four possible determinants for increased effectiveness. The applications were categorized into mechanisms applied based on commonalities between PROMs interventions. Subsequently, we captured a broader perspective by determining the impact of PROMs interventions by the medical domain, the type of PROM used in the intervention, and by the separate outcome dimensions used to measure the effect of the intervention. For the latter, we decided to only present those which were measured in at least 3 studies. We discerned studies which used the same PROM outcome as in the intervention from studies which (only) used different outcomes. Finally, for each determinant and outcome dimension, the average quality of studies was calculated.

## Results

**Fig. 1:**
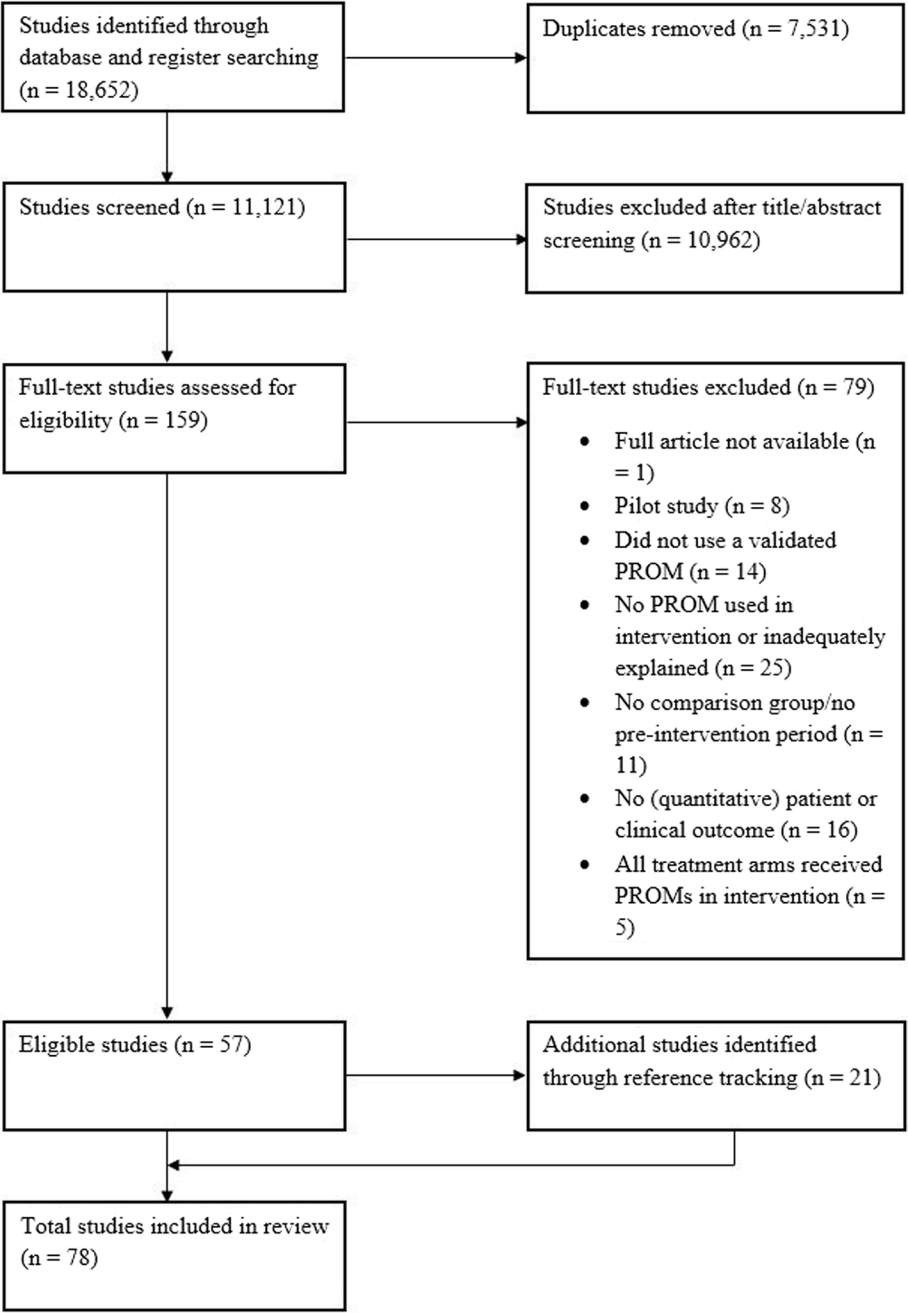
PRISMA flowchart of study selection

The PRISMA diagram depicting the selection process is presented in Fig. 1. A total of 18,652 records were identified. After removing duplicates, 11,121 records were screened at title-abstract level, of which 159 were screened at full-text; 57 records were found to be eligible for inclusion [17, 19, 28, 34–88]. Through reference tracking another 21 records were identified [17, 89–108], leading to a total of 78 included studies. Two studies presented outcomes in two separate publications; these were combined resulting in 76 unique studies [17, 74, 75, 87].

### Study characteristics

An overview of study characteristics, PROMs used, overall study impact and quality is presented in Table 1 (micro-level) and Table 2 (meso-/macro-level). Below we shortly describe the included studies: for a more detailed description of study characteristics refer to Supplementary Material 2, and for extended tables of study characteristics, quality assessment and outcomes extracted refer to Supplementary Material 3.

**Table 1:** Study characteristics at the micro-level (sorted by medical domain)

Author (year)	Domain	Patients	Number of patients (I = Intervention, C = Control)	PROM(s) used (GEN = generic, DS = disease-specific)	Overall impact	Quality of study
**Decision-aid**						
Jayakumar (2021)	Orthopedics	Considering knee replacement for OA	I:69; C:60	KOOS-JR (DS), PROMIS Global 10 (GEN)	+	Moderate
Bansback (2022)	Orthopedics	Considering knee replacement for OA	I:82; C:81	EQ-5D-5L (GEN)	+	Weak
Volkmann (2015)	Rheumatology	Considering knee replacement for OA	I:111; C:NA	WOMAC (DS)	+	Moderate
**Feedback to patient**						
Ngo (2022)	Gyneacology	Pregnant women	I:89; C:103	PUQE (DS)	~	Moderate
Rogers (2021)	Oncology	Head-neck cancer, curatively treated	I:140; C:148	PCI Head Neck Cancer (DS)	+	Moderate
Steele Gray (2021)	Primary care	Elderly	I:23; C:21	PROMIS Global Health Scale (GEN), Pain Interference (DS), HAQ (GEN)	~	Weak
Berdal (2023)	Rehabilitation	Rheumatic or musculoskeletal diseases	I:168;C:206	PSFS (DS), EQ-5D-5L (GEN), EQ VAS (GEN), 30 s sit-to-stand test	~	Moderate
Gossec (2016)	Rheumatology	Reumatoid arthritis	I:159; C:161	RAPID-3/HAQ2 (GEN), RAID (DS), symptoms as free text	~	Moderate
**Feedback to provider**						
Hadjistavropoulos (2009)	Community care	Elderly, with complex medical problems	I:88; C:56	Pain Assessment Battery (DS), GPM (DS), GDS short form (DS), pain drawing	~	Weak
Almario (2016)	Gastroenterology	Gastrointestinal disease	I:217; C:154	PROMIS Gastrointestinal (DS)	~	Moderate
Kjaer (2016)	Oncology	Head-neck cancer, finished treatment	I:132; C:134	HADS (DS), symptoms relevant to head-neck cancer survivors	+	Weak
Velikova (2004)	Oncology	Any cancer, commencing treatment	I:144, 70; C:72	EORTC-QLQ-C30 (DS), and HADS (DS)	+	Weak
Detmar (2002)	Oncology	Any cancer, undergoing palliative chemotherapy	I:114; C:100	EORTC-QLQ-C30 (DS)	~	Weak
Rosenbloom (2007)	Oncology	Advanced breast, lung or colorectal cancer	I:71, 73; C:69	FACT-G (DS), including relevant cancer-type subscale	~	Strong
Hilarius (2008)	Oncology	Any cancer, undergoing adjuvant or palliative chemotherapy	I:111; C:108	EORTC-QLQ-C30 (DS), including relevant cancer-type (breast (QLQ-BR23), colorectal (QLQ- CR38), or lung cancer (QLQ-LC13))	~	Strong
Barbera (2015)	Oncology	Stage I–III breast cancer, received adjuvant chemotherapy	I:2541; C:5818	ESAS (DS)	+	Strong
Patel (2022)	Oncology	Advanced cancer	I:64; C:64	ESAS (DS)	+	Strong
Skovlund (2021)	Oncology	Metastic melanoma	I:137; C:142	EORTC-QLQ-C30 (DS), HADS (DS)	+	Weak
Ackermans (2017)	Orthopedics	Hip or knee OA	I:72; C:70	HOOS-PS/KOOS-PS (DS), NRS Pain (DS)	+	Strong
Holm (2020)	Orthopedics	Hip or knee OA	I:6245; C:N/A	NRS pain (DS), HOOS/KOOS (DS), EQ-5D (GEN), PSFS (DS), physical activity (DS), OA-QI (DS), ASES (DS)	+	Strong
De Wit (2008)	Pediatrics	Diabetes	I:46; C:45	PedsQL (GEN), Generic and Diabetes module (DS)	+	Moderate
Fihn (2004)	Primary care	Veterans	I:5801; C:3218	SF-36 (GEN), Seattle Outpatient Satisfaction Questionnaire (PREM), 1 of 6 disease-specific questionnaires (Seattle Angina, Seattle Obstructive Lung Disease, Drinking Practices, Seattle Diabetes, Seattle Hypertension, HSCL)	~	Weak
Kroenke (2018)	Primary care	Visiting for any reason	I:151; C:149	SPADE symptoms (GEN)	~	Moderate
Reiber (2004)	Primary care	Veterans	I:3701; C:2020	Seattle Diabetes Questionnaire, SF-36 (GEN), SOSQ (PREM)	~	Weak
Richardson (2008)	Primary care	Elderly	I:134; C:131	Self Report Task Modification and Disability Scale (DS), HUI (GEN), SF-36 (GEN), several physical and functional performance measures	+	Moderate
Santana (2010)	Pulmonary medicine	Pre- and post-lung transplant	I:108; C:105	HUI2/3 (GEN)	~	Moderate
**Monitoring**						
Davidson (2010)	Cardiology	Myocardial infarction	I:80; C:77	PHQ-9 (DS)	+	Moderate
de Jong (2017)	Gastroenterology	IBS	I:465; C:444	Monitor IBD At Home (DS)	+	Moderate
Berinstein (2022)	Gastroenterology	IBS	I:100; C:105	CD-PRO (DS), UC-PRO (DS)	+	Strong
Cross (2019)	Gastroenterology	IBS	I:115, 116; C:117	HBI (DS), SSCAI (DS)	+	Moderate
Pooni (2023)	General surgery	Colorectal surgery	I:128; C:125	QoR-15 (DS)	+	Strong
Girgis (2009)	Oncology	Non-localized breast and colorectal cancer	I:120, 119; C:117	HADS (DS), EORTC-QLQ-C30 (DS), SCNS short form (DS), selected items from the NA-ACP (DS)	~	Moderate
Cooley (2022)	Oncology	Lung cancer	I:89; C:91	PHQ-9 (DS), SDS (DS), FACT-G (DS), AUDIT (DS), MSAS (DS)	~	Weak
Price (2023)	Oncology	Cancer patients with depression	I:165; C:NA	PHQ-9 (DS) and GAD-2 (DS)	+	Weak
Livanainen (2023)	Oncology	Colorectal cancer, undergoing chemotherapy	I:36; C:35	NCTCAE (DS)	~	Weak
Basch (2016)	Oncology	Metastatic breast, genitourinary, gynecologic or lung cancer	I:441; C:325	NCTCAE (DS)	+	Moderate
Sharpe (2014)	Oncology	Cancer patients with depression	I:253; C:247	PHQ-9 (DS)	+	Weak
Patel (2020)	Oncology	Hematologic or advanced stage cancer	I:186; C:102	Edmonton Symptom Assessment Scale (DS)	+	Strong
Maguire (2021)	Oncology	Non-metastatic breast, colorectal, (non-)Hodgkin’s cancer, undergoing chemotherapy	I:415; C:414	DCTAQ (DS)	+	Strong
Epstein (2007)	Pediatrics	ADHD	I:162; C:215	CPRS (DS), CTRS (DS)	~	Moderate
Dobscha (2006)	Primary care	Moderate to severe depression	I:189; C:186	PHQ-9 (DS)	+	Strong
Balestrieri (2020)	Primary care	Depression	I:66; C:32	PHQ-9 (DS) and IDS-SR (DS)	+	Moderate
Dhingra (2021)	Primary care	Chronic pain	I:256; C:272	FPS (DS), SBIRT (DS), PHQ-2 (DS), BPI short form (DS), PROMIS Pain Interference short form (DS)	~	Weak
Katon (1996)	Primary care	Depression	I:77; C:76	BDI short form (DS)	+	Moderate
Katzelnick (2000)	Primary care	Depression	I:218; C:189	HAM-D (DS)	+	Moderate
Unützer (2002)	Primary care	Depression	I:218; C:189	PHQ-9 (DS)	+	Weak
Simon (2011)	Primary care	Depression	I:106; C:102	PHQ-9 (DS)	+	Weak
Katon (2004)	Primary care	Diabetes patients, with depression	I:164; C:165	PHQ-9 (DS)	+	Weak
Carola Pèrez (2021)	Psychiatry	Depression	I:84; C:83	PHQ-9 (DS)	~	Moderate
Tirelli (2020)	Reumatology	Juvenile Idiopathic Arthritis, subtype Enthesitis-Related Arthritis	I:54; C:98, 51	JADAS (DS), PROMIS physical function (DS), NRS pain (DS)	~	Moderate
Buckley (2020)	Rheumatology	Juvenile Idiopathic Arthritis	I:97; C:NA	JADAS (DS), PROMIS physical function (DS), NRS pain (DS)	+	Moderate
**No feedback**						
Baker (2023)	Dermatology	Eczema	I:147; C:149	POEM (DS)	+	Moderate
McCambridge (2007)	General public	University students	I:217; C:204	AUDIT (DS)	+	Moderate
Jaensson (2017)	General surgery	Various types of day surgery	I:513; C:514	SwQoR (DS)	+	Strong
**Screening**						
Frasure-Smith (1997)	Cardiology	Myocardial infarction	I:692; C:684	GHQ (DS)	~	Strong
Kronish (2020)	Cardiology	Myocardial infarction	I:499, 501; C:500	PHQ-8 (DS)	~	Weak
Allen (2011)	Community care	War veterans	I:97; C:NA	DASS (DS), AUDIT (DS)	+	Weak
van der Zee-van den Berg (2017)	Midwife care	Mothers who just gave birth	I:1843; C:1246	EPDS (DS)	+	Moderate
Ferrell (2021)	Oncology	Palliative cancer, undergoing a therapeutic clinical trial	I:239; C:240	Distress Thermometer (DS), FACT-G (DS)	+	Weak
Shyu (2013)	Orthopedic and trauma surgery	Hip fracture patients	I:99; C:101, 99	GDS short form (DS)	+	Strong
Mallen (2017)	Primary care	Hip or knee osteoarthritis patients	I:1339; C:703	GAD-2 (DS), PHQ-2 (DS), NRS pain (DS)	-	Moderate
Fortmann (2020)	Primary care	Diabetes patients	I:236;C:239	PHQ-9 (DS)	+	Moderate
Rollman (2021)	Cardiology	Heart failure	I:251; C:252, 126	PHQ-2 and -9 (DS)	+	Moderate
Regueiro (2019)	Gastroenterology	IBS	I:322; C:NA	Harvey-Bradshaw Index (DS) for CD or Ulcerative Colitis Activity Index for UC, Short Inflammatory Bowel Disease questionnaire (DS), PHQ-9 (DS), GAD-7 (DS)	+	Moderate
Howell (2020)	Oncology	Various types of cancer	I:13,260, 10,875; C:57,594, 48,068	ESAS (DS), DART (DS), BPI (DS), CFS (DS), GAD (DS), PHQ (DS), occasionally the CPC (DS) or SDI (DS)	+	Strong
Wylde (2022)	Orthopedics	Received knee replacement surgery	I:242; C:121	BPI (DS), HADS (DS), PainDETECT (DS), Douleur Neuropathique 4 (DS)	+	Weak
Buxton (2012)	Primary care	Low income and uninsured	I:36; C:81	SF-12 (GEN), AUDIT (DS), PHQ-9 (DS)	~	Moderate
Wu (2018)	Primary care	Diabetes type 2	I:366, 380; C:341	PHQ -2 and/or -9 (DS)	+	Strong

Studies are categorized according to the mechanism applied. Additional study characteristics, full quality assessment and all extracted outcomes can be found in Supplementary Material 3, Table 1 to 3

*ADHD* Attention Deficit Hyperactivity Disorder, *ASES* Arthritis Self-Efficacy Scale, *AUDIT* Alcohol Use Disorders Identification Test, *BDI* Beck Depression Inventory, *BPI* Brief Pain Inventory, *CD-PRO* Crohn’s Disease PRO, *CFS* Cancer Fatigue Scale, *COOP* Primary Care Cooperative Information Project, *CPC* Canadian Problem Checklist, *CPRS* Conners Parent Rating Scale, *CTRS* Conners Teachers Rating Scale, *DART* Distress Assessment Response Tool, *DASS* Depression Anxiety Stress Scales, *DCTAQ* Daily Chemotherapy Toxicity Self-Assessment Questionnaire, *EORTC-QLQ-C30* The European Organization for Research and Treatment of Cancer quality of life questionnaire, *EPDS* Edinburgh Postnatal Depression Scale, *EQ-5D-5L* Five-level version of the EQ-5D, *ESAS* Edmonton Symptom Assessment System, *FACT-G* Functional Assessment of Cancer Therapy—General, *FPS* Faces Pain Scale, *GAD* Generalized Anxiety Disorder, *GDS* Geriatric Depression Scale, *GHQ* General Health Questionnaire, *GPM* Geriatric Pain Measure, *HADS* Hospital Anxiety and Depression Scale, *HAM-D* Hamilton Depression Rating Scale, *HAQ* Health Assessment Questionnaire, *HBI* Harvey-Bradshaw Index, *HOOS-PS* Hip disability and Osteoarthritis Outcome Score – Physical Function, *HSCL* Hopkins Symptom Checklist, *HUI* Health Utilities Index Mark, *IBS* Inflammatory Bowel Disease, *IDS-SR* Inventory of Depressive Symptomatology – Self Rated, *JADAS* Clinical Juvenile Arthritis Disease Activity Score, *KOOS-JR* Knee Injury and Osteoarthritis Outcome Score for Joint Replacement, *KOOS-PS* Knee disability and Osteoarthritis Outcome Score – Physical Function, *MSAS* Memorial Symptom Assessment Scale, *NA-ACP* Needs Assessment for Advanced Cancer Patient Questionnaire, *NCTCAE* National Cancer Institute's Common Terminology Criteria for Adverse Events, *NRS* Numerical Rating Scale, *OA-QI* OsteoArthritis Quality Indicator Questionnaire, *OHS* Oxford Hip Score, *OKS* Oxford Knee Score, *PCI* Patient Concerns Inventory, *PedsQL* Pediatric Quality of Life Inventory, *PHQ* Patient Health Questionnaire, *POEM* Patient Oriented Eczema Measure, *PREMs* patient-reported experience measures, *PROMs* patient-reported outcome measures, *PROMIS* Patient-Reported Outcomes Measurement Information System, *PSFS* Patient Specific Functional Scale, *PUQE* Pregnancy Unique Quantification of Emesis, *QoR-15* Quality of Recovery questionnaire, *RAID* Reumatoid Arhritis Impact of Disease, *RAPID-3/HAQ2* RAPID3 Health Assessment Questionnaire, *SBIRT* Screening, Brief Intervention, and Referral to Treatment questionnaire, *SCNS* Supportive Care Needs Survey, *SDI* Social Difficulties Inventory, *SDS* Symptom Distress Scale, *SF-36* Short Form Health Survey, *SOSQ* Seattle Outpatient Satisfaction Questionnaire, *SPADE* Sleep, pain, anxiety, depression, and low energy/fatigue, *SSCAI* Simple Clinical Colitis Activity Index, *SwQoR* Swedish Quality of Recovery Scale, *UU-PRO* Ulcerative Colitis PRO, *VAS* Visual Analogue Scale, *WOMAC* Western Ontario and McMaster Universities Osteoarthritis Index

**Table 2:** Study characteristics at the meso-/macro-level (sorted by medical domain)

Author (year)	Domain	Patients	Number of patients	PROM(s) used (GEN = generic, DS = disease-specific)	Overall impact	Quality of study
**Benchmarking**						
Boyce (2015)	Orthopedics	Receiving primary hip arthroplasty	I:230; C:228	OHS (DS)	~	Weak
Varagunam (2014)	Orthopedics and general surgery	Receiving hip or knee arthroplasty, varicose vein or inguinal hernia surgery	I: 7 k-30 k; C:NA	EQ-5D-5L (GEN), EQ VAS (GEN), 1 of 3 disease-specific questionnaires (OHS, OKS, AVVQ)	~	Moderate
Weingarten (2000)	Primary care	Elderly	I:541; C:543	Dartmouth COOP (GEN)	~	Moderate
Kumar (2021)	Urology	Undergoing prostate surgery for cancer	I:212; C:210	Selected items (continence, sexual function) from the EPIC (DS)	-	Weak
**In-depth analysis of data**						
Haller (2011)	Internal and surgical departments	Wards	I:1237; C:1113	IPO questionnaire (DS), NRS pain	+	Moderate
Zaslansky (2019)	Surgical departments	Wards	I:? C:?	IPO questionnaire (DS), NRS pain	+	Weak
Garduño-López (2021)	Surgical departments	Wards	I:? C:?	IPO questionnaire (DS), NRS pain	+	Weak
**PDCA-cycle**						
Partridge (2016)	Orthopedics	Patient receiving total knee arthroplasty	I:827, C:441	OKS (DS), EQ-5D-3L (GEN)	+	Moderate

Studies are categorized according to the mechanism applied. Additional study characteristics, full quality assessment and all extracted outcomes can be found in Supplementary Material 3, Table 1 to 3

*AVVQ* Aberdeen Varicose Vein Questionnaire, *COOP* Primary Care Cooperative Information Project, *EPIC* Expanded Prostate Cancer Index Composite, *EQ-5D-3L* Three-level version of the EQ-5D, *EQ-5D-5L* Five-level version of the EQ-5D, *IPO* International Pain Outcomes Questionnaire, *NRS* Numerical Rating Scale, *OHS* Oxford Hip Score, *OKS* Oxford Knee Score, *VAS* Visual Analogue Scale

#### Micro-level

Sixty-eight out of 76 studies provided evidence on the use of PROMs at the micro-level [17, 19, 28, 34–36, 38–44, 46–48, 50–56, 58, 59, 62–71, 73–93, 95–108]. Most studies were conducted in the United States (n = 32), and were in the medical domains primary care (n = 17), oncology (n = 19), gastroenterology (n = 5) and orthopedic (trauma) surgery (n = 6). Fifty-five studies used a disease-specific instrument in their intervention, 3 used a generic instrument and 10 a combination. Sixteen studies were of strong quality, 31 were of moderate quality and 21 were of weak quality.

#### Macro-level

Eight out of 76 studies provided evidence of the use of PROMs at the macro-level [37, 45, 49, 57, 60, 61, 72, 94], and no studies were found at the meso-level. Studies were conducted in various countries. Most studies were conducted in surgical fields (n = 7), of which 3 in both non-surgical and surgical fields; the eighth study was conducted in primary care. Five studies used a disease-specific PROM, 1 used a generic PROM, and 2 used a combination. Four studies were rated as moderate quality, while the other 4 were rated as weak quality.

### Impact by determinants and outcome dimensions

Outcome of PROMs interventions by determinants are summarized in Table 3 (micro-level) and Table 4 (meso-/macro-level). Table 5 shows the impact by outcome dimensions. The quality of studies for each determinant generally indicated “moderate” quality, both at the micro- and meso-/macro-level; the exception is highlighted. Six mechanisms were identified at the micro-level, and 3 at the meso-/macro-level.

**Table 3:** Overall impact by determinants at the micro-level

		**Number of studies**	**Improvement (%)**	**Quality of studies (average)**
**Mechanism**	Feedback to patient	5	1 (20)	1.8
	Feedback to provider	18	9 (50)	1.9
	Screening	14	10 (71)*	2.0
	Monitoring	25	18 (72)	1.9
	No feedback	3	3 (100)	2.3
	Decision-aid	3	3 (100)	1.7
**Medical domain**	Cardiology	4	2 (50)	2.0
	Community care	2	1 (50)	1.0
	Dermatology	1	1 (100)	2.0
	Gastroenterology	5	4 (80)	2.2
	General public	1	1 (100)	2.0
	General surgery	2	2 (100)	3.0
	Gyneacology	1	0 (0)	2.0
	Midwife care	1	1 (100)	2.0
	Oncology	19	13 (68)	1.9
	Orthopedics/trauma surgery	6	6 (100)	2.2
	Pediatrics	2	1 (50)	2.0
	Primary care	17	10 (59)*	1.7
	Psychiatry	1	0 (0)	2.0
	Pulmonary medicine	1	0 (0)	2.0
	Rehabilitation	1	0 (0)	2.0
	Rheumatology	4	2 (50)	2.0
**Type of PROM**	Disease-specific	55	39 (71)*	2.0
	Generic	4	1 (25)	1.5
	Combination	9	4 (44)	1.9

^*^ One study showed a deterioration

*PROM* Patient-Reported Outcome Measure

**Table 4:** Overall impact by determinants at the meso-/macro-level

		**Number of studies**	**Improvement (%)**	**Quality of studies (average)**
**Mechanism**	Benchmarking	4	0 (0)*	1.3
	In-depth analysis of data	3	3 (100)	1.7
	PDCA-cycle	1	1 (100)	2.0
**Medical domain**	Orthopedics	2	1 (50)	1.7
	Primary care	1	0 (0)	1.0
	Urology	1	0 (0)*	2.0
	Various internal and surgical departments	4	3 (75)	1.5
**Type of PROM**	Disease-specific	5	3 (60)*	1.4
	Generic	1	0 (0)	1.0
	Combination	2	1 (50)	2.0

^*^ One study showed a deterioration

*PROM* Patient-Reported Outcome Measure

**Table 5:** Impact by outcome dimensions

		**Micro-level**			**Meso-/macro-level**	
	**Number of studies**	**Improvement (%)**	**Quality of studies (average)**	**Number of studies**	**Improvement (%)**	**Quality of studies (average)**
**PROMs**						
Functioning						
Physical	29	12 (41)	1.9	2	0	1.0
Mental	25	8 (32)	1.8	1	0	1.0
Social	16	6 (38)	1.8	1	0	1.0
HRQoL	29	11 (38)	1.8	5	2 (40)	1.6
Role limitations						
Physical	5	1 (17)	2.0	-	-	-
Emotional	5	0	2.0	-	-	-
General health perceptions	8	6 (75)	2.4	1	0	1.0
Symptoms combined	46	26 (57)**	1.9	5	3 (60)**	1.6
Depression	25	14 (56)	1.8	2	0	2.5
Anxiety	14	6 (43)	2.0	2	1 (50)	2.5
Alcohol use/disorder	3	1 (33)	1.7	-	-	-
Pain	17	7 (42)**	1.9	5	2 (40)	1.6
Vitality/fatigue	5	3 (60)	1.8	1	0	3.0
Nausea	4	0	2.3	2	1 (50)	1.0
Decision-conflict and readiness	4	3 (75)		-	-	-
**PREMs**						
Satisfaction	23	10 (43)	2.0	-	-	-
Patient-physician relationship	5	1 (20)	2.0	-	-	-
Experience with care	7	4 (57)	2.0	-	-	-
Supportive needs	3	1 (33)	2.7	-	-	-
Patient-activation	7	4 (57)	2.1	-	-	-
Physician awareness of HRQoL	2	0	2.0	-	-	-
**Clinical outcomes**						
Complications*	8	1 (13)	1.8	2	0	1.5
(Re)admissions	17	5 (29)	2.2	-	-	-
Emergency department visits	12	7 (58)	2.4	-	-	-
Survival	5	0	2.0	-	-	-
Lab values	4	2 (50)	2.0	-	-	-
**Outcome same as PROM used in intervention**	32	18 (56)**	1.8	7	3 (42)**	1.4
**Outcome not the same as PROM used in intervention**	36	26 (72)	2.0	1	1 (100)	2.0

*Complications also vary by domain and intervention, e.g., a bleed in myocardial infarction patients

**One study showed a deterioration

*PROM* Patient-Reported Outcome Measure; *PREM* Patient-Reported Experience Measure, *HRQoL* Health-Related Quality of Life

### Impact by mechanism

#### Micro-level

##### Feedback of PROMs to patient

One of 5 studies employing feedback of PROMs to patients fed back (raw) scores directly [54], 3 included a graphical display of PROMs scores [55, 78, 85], and 1 combined a narrative report with a graphical display [43]. Studies were conducted in various domains. One (20%) study conducted in head-cancer patients fed back data from a comprehensive inventory of disease-related symptoms and found an improved overall outcome, driven by improved symptoms (pain and activity), mental and physical functioning [54].

##### Feedback of PROMs to provider

Two of the 18 studies employing feedback of PROMs to providers used (raw) scores in their report [79, 90], 4 included a narrative report [52, 53, 73, 93], 8 included a graphical display [17, 36, 44, 47, 48, 84, 91, 92], and 3 combined a narrative report with a graphical display [34, 41, 89]. Overall, nine (53%) studies found an improvement in outcome [17, 34, 47, 53, 73, 84, 89, 90].

When looking at the information collected, 14 of 18 studies fed back PROMs to patients which covered disease-specific information such as hip functioning, cancer-related, or gastrointestinal symptoms [17, 34, 36, 41, 47, 53, 73, 79, 84, 89–93]. Of these 14 studies, 9 (64%) found an improvement in outcome [17, 34, 47, 53, 73, 79, 84, 89, 90]. Most studies pertained to cancer-related symptoms (n = 8) of which 5 (63%) reported an improvement via various outcome dimensions, including reduced emergency department (ED) visits or readmissions (n = 2), improved physical, mental and social functioning (n = 1), symptoms (depression and cancer-related) (n = 1) or experience with care (n = 1) [17, 47, 79, 84, 89]. The remaining 4 studies fed back PROMs to the provider pertaining to general HRQoL and/or pain, and found no improvement in outcome [44, 48, 52, 55].

##### Using PROMs to screen for disease or symptoms

Seven studies out of 14 used PROMs to screen for depression [28, 35, 50, 56, 71, 98, 102], and 1 study for oncological symptoms [70], to initiate treatment or a care pathway. Of these, five (63%) studies observed an improved outcome driven by improved symptoms (depression, stress or anxiety) (n = 4), improved mental (n = 2), social (n = 2), and physical functioning (n = 1), and reduced ED visits and readmissions (n = 1) [28, 35, 56, 70, 71]. One study found an outcome deterioration via worsened pain symptoms [50].

Six studies combined the screening for depression with follow-up monitoring to evaluate whether the treatment works, and potentially adjust if treatment was ineffective [38, 59, 74, 83, 88, 105]. Of these, three also incorporated disease-specific information: knee functioning [88], cancer-related [74], and gastro-intestinal symptoms [105]. Five (83%) out of 6 studies found improved outcome particularly via improved symptoms (depression and anxiety) (n = 4) and reduced ED visits (n = 2) [59, 74, 83, 88, 105]. Two of three disease-specific symptoms also improved, except for oncological symptoms [74].

##### Using PROMs to monitor symptoms

Twelve out of 25 studies used PROMs to identify patients under treatment exceeding predefined thresholds of symptoms and linked these to treatment changes, increased monitoring or care pathways [39, 63, 66, 67, 81, 86, 95, 97, 100, 103, 107, 108]; 10 (83%) found an improved outcome [39, 63, 66, 81, 95, 97, 100, 103, 107, 108]. Seven studies also used PROMs monitor treatment but did not explicitly mention the use of predefined algorithms [40, 42, 69, 82, 99, 101, 104]; 4 (57%) reported an improvement [82, 99, 101, 104]. Six studies incorporated PROMs into the clinical pathway and sent out alerts upon exceeding a threshold without specific guidance to the provider [64, 68, 76, 80, 96, 106], 1 of these also used PROMs to monitor treatment response [106]; three (50%) found an improved outcome [64, 96, 106].

When looking at the information collected, 13 out of 25 studies used PROMs to monitor existing depression symptoms [42, 63, 68, 69, 80, 82, 97, 99–101, 106–108]. Of these, 10 (77%) found an improved outcome, mostly driven by improved depression symptoms (n = 9) and satisfaction (n = 5) [63, 69, 82, 97, 99–101, 106–108]. Five studies used PROMs to monitor cancer-related symptoms [64, 67, 76, 103, 104], of which 3 (60%) found various improved outcomes including HRQoL, physical and mental functioning, and satisfaction [64, 103, 104]. Three studies monitored gastro-intestinal symptoms in patients with inflammatory bowel disease and all (100%) found reduced readmissions (n = 2) and improved HRQoL (n = 1) [39, 66, 96]. The remaining 4 studies were conducted in various domains [40, 81, 86, 95], of which two showed improved outcomes. The first monitored surgical recovery in colorectal surgery patients and found improved perception of general health, anxiety and satisfaction. The other used PROMs to guide treatment in children with juvenile idiopathic arthritis and found reduced pain and arthritis activity [81, 95].

##### No feedback: filling out effect of PROMs

One of 3 studies tested the hypothesis of whether merely filling out alcohol abuse PROMs would reduce alcohol use by a direct measurement effect [51]. Similarly, another study collected PROMs weekly in patients with eczema without any additional interventions [62]. The third study collected PROMs daily after surgery via an app; patients could always contact their provider via the e-portal [46]. All (100%) studies reported improved outcome due to improved symptoms (depression and alcohol dependency) (n = 2) and improved HRQoL (n = 1).

##### PROMs in decision-aids

In three studies a one-time PROM was used in a decision-aid along an education component to help with treatment choice (surgical vs. conservative) in patients with knee osteoarthritis [19, 58, 77]. All studies (100%) found an improvement in shared-decision making, while 1 of these only found this effect in females [58].

#### Meso-/macro-level

##### PROMs in benchmarking

Three benchmarking studies used case-mix adjusted PROM scores [37, 49, 57], while the fourth used unadjusted scores [94]. Three studies presented performance reports to the provider, which included PROM scores and how they compared to peer providers [37, 49, 94]; in 2 studies complication rates were also presented [37, 49]. The other study evaluated a nationwide PROMs collection program, which provided both patients and providers the option to check providers' PROMs outcomes [57]. All studies were of weak quality, and did not find an improvement in outcome; 1 study even reported a potential worsening [49].

##### PROMs in in-depth analysis of data

Three studies used PROM data in combination with guidelines, teaching and protocols to improve pain management in various surgical and non-surgical departments [45, 60, 72]. One of these studies also used a feedback loop by a department representative to evaluate and provide advice on the implemented initiatives [45]. The two other studies pertained to the same quality initiative aimed to reduce the pain of patients admitted to hospitals but were conducted in different developing countries/departments [60, 72]. All 3 (100%) studies found an improvement in outcome due to reduced pain (n = 3) and nausea (n = 2) symptoms in particular.

##### PROMs in PDCA-cycles

One study conducted a PDCA-cycle where they introduced an improved total knee implant and changed their surgical technique, guided by and evaluated with PROMs scores [61]: an overall improvement in outcome (HRQoL) was observed.

### Impact by medical domain

#### Micro-level

At the micro-level, the medical domains in which PROM interventions were conducted which seemed to be consistently associated with improved outcome were orthopedic (trauma) surgery (n = 6 studies, 100% effective), gastroenterology (n = 5, 80%), oncology (n = 19, 68%), and primary care (n = 17, 59%). Less effective seemed cardiology (n = 4, 50%) and rheumatology (n = 4, 50%). Limited evidence was available for other domains.

#### Meso-/macro-level

Interventions conducted in orthopedics, primary care, and urology were not found to be related to improved outcome. Four studies covered various internal and surgical departments, of which 3 (75%) showed improved outcome.

### Impact by type of PROM used in intervention

#### Micro-level

Most studies used a disease-specific PROM, which showed the highest percentage of improved outcomes (n = 55 studies, 71% effective). Generic PROMs or a combination of both showed an overall lower percentage (n = 13, 38%). While disease-specific PROMs were used in all mechanisms, generic PROMs were used in studies employing the “feedback” mechanism (n = 10), “decision-aids” (n = 2), and once (combined with a disease-specific PROM) in “screening”.

#### Meso-/macro-level

According to the type of PROM (disease-specific vs. generic) no specific pattern was observed.

### Impact by outcome dimensions

#### Micro-level

In this section, we describe the impact of the PROMs-interventions on the outcome dimensions (PROMs, PREMs or clinical outcomes), regardless of the mechanism or other determinants.

Regarding PROMs, studies often showed an improvement in general health perceptions (n = 8 studies, 75% effective), decision-readiness and conflict (n = 4, 75%) and symptoms overall (n = 46, 57%). Particularly depression was evaluated often (n = 25), and improved in 57% of studies. The percentage decreased for HRQoL (n = 29, 38%) and physical and mental functioning domains.

Regarding PREMs, satisfaction was most often studied (n = 23), and improved in less than half of studies (43%). Patient-activation and experience with care tended to improve slightly more often (n = 7, 57%, for both outcomes).

As for clinical outcomes, twelve studies analyzed emergency department visits, of which 58% found an improvement. Fewer studies observed a positive effect on complications (n = 8, 13%) and (re)admissions (n = 17, 29%), and no studies observed an effect on survival (n = 5, 0%).

Studies which used a different outcome than the PROM in the intervention more often had an improved overall outcome (n = 36, 72%), compared to those which did not (n = 32, 56%).

#### Meso-/macro-level

With regard to PROMs, symptoms showed improved most often, which mostly pertained to pain (n = 5, 60%). HRQoL was also measured in 5 studies, however, improved in less studies (40%). Other domains and outcomes were studied in only a few studies, and showed no improvement.

## Discussion

In this systematic review, evidence on the use of PROMs to improve patient-related outcomes at the micro- (68 studies) and meso-/macro- [8] levels was collected and analyzed. Moreover, determinants for increased effectiveness were elucidated.

At the micro-level, 44% of studies employing direct feedback of PROMs to the provider and/or patient resulted in improved patient outcomes, which is in line with previous reviews [8–15]. A contemporary development was to use PROMs to screen for disease or to monitor existing disease. These studies linked the PROMs scores to care pathways or treatment adaptations, and approximately 70% of studies found improved outcomes. This approach was particularly effective for depression, oncological and gastroenterological disease. A novel application was to use PROMs to inform patients considering knee arthroplasty, which generally resulted in improved decision-quality. At the meso-/macro-level, current evidence does not support using PROMs in benchmarking. The scarce evidence available suggests, however, that PROMs might be of value in an in-depth analysis of the performance of departments and hospitals and PDCA-cycles. At both the micro- and meso-/macro-level, studies more often employed disease-specific PROMs, which – in comparison with studies which employed generic PROMs – found improved outcomes more often.

The evidence at all levels was of moderate quality at best, which raises concerns regarding the validity of the findings.

### Micro-level

Providing feedback on the PROM scores to patients or providers is generally thought to benefit outcomes via improved patient-healthcare professional communication and identification of problematic symptoms [16]. This application is often used in patients with chronic disease who have multiple visits to their doctor, which in our review included diabetes, gastrointestinal disease, oncology, orthopedics, transplantation care; most evidence was available for oncology [8, 27]. For example, two studies applied a tailored symptom inventory for head-neck cancer patients and found a positive impact on PROMs [47, 54]. The effectiveness may be because this group presumably experiences a number of severe physical symptoms (e.g., problems with swallowing) which, if timely detected, are sensitive to treatment.

The application of PROMs to improve patient outcomes seems particularly effective if a deviation from the acceptable threshold occurs and can be linked to a recognizable action by the clinician, such as referral or treatment adaptation. This mechanism was effective in several studies in the medical domains, including depression, oncology and gastrointestinal care. For example, monitoring patients with diagnosed diseases such as inflammatory bowel disease or screening for disease with an expected high burden in the studied population such as post-partum depression may be beneficial [28, 39]. The purpose and goal of the tool may be clearer for both patient and provider, which could increase its effectiveness.

Various reasons may underlie decreased effectiveness of PROM-interventions. Firstly, a general trend was observed that studies utilizing generic PROMs found less positive effect overall, and these studies mostly did not link a generic PROM to a care pathway (such as “screening” or “monitoring”). Generic PROMs may provide insufficient insight into treatable or modifiable factors related to the studied population. However, it should be noted, one of the identified decision-aids successfully employed only a generic measure in patients considering knee arthroplasty [19]. Combined, we believe this underlines the fact that the choice of PROM in the intervention should be driven by the intended use. Secondly, the measured outcome may play a role: PROM interventions tended to have a more pronounced impact on general health perceptions and symptom burden, but less so on certain outcomes such as HRQoL in general or survival. Other reasons for failure may include patients’ resistance to discussing symptoms, time constraints in clinical practice and lack of provider continuity, and implementation hurdles through lack of knowledge [16].

The evaluation of interventions based on systematic PROM feedback appears to be a challenge. Firstly, the definition of 'control' treatment: about a third of the studies collected PROMs in the control group, unconnected to feedback or another intervention. This may decrease the difference as the collection of PROMs itself may induce beneficial effects as observed in 3 studies [46, 51, 62]. These findings suggest a Hawthorne-like effect through the completion of PROMs alone [51, 109]. The patient’s self-knowledge and awareness are increased, and filling out the questionnaire may increase their empowerment to take a more active role in their healthcare [34]. We expected this effect to be relatively limited, as approximately half of studies used a different outcome measure than the PROM in the intervention and generally found an improvement. Secondly, most studies did not measure intervention compliance making it impossible to know to what extent (and how) patients or providers used the PROM interventions. Thirdly, PROMs are generally part of a more complex intervention with multiple facets (e.g., patient education), and it is impossible to isolate the exact role of the PROM in the intervention. However, we believe this is also one of the key roles of PROMs in contemporary medicine; they can enhance interventions by offering important insight into patient outcomes.

### Meso-/macro-level

The 4 studies which evaluated PROM benchmarking did not find a benefit. Multiple reasons for the intervention not being successful have been suggested. Boyce et al. noted that PROMs have not been developed nor validated as performance measures, and the choice of PROM may play a role in the usability of the provided feedback [37]. It is possible that inter-provider comparisons do not inherently motivate professionals to initiate additional audits and research activities or professionals may lack the knowledge to undertake such initiatives. The included studies do not describe how the data was (or wasn’t) used in a feedback process of change. Kumar et al. suggested that further improvement might be prevented when the quality of care is already high [49]. The quality of the benchmarking process is also dependent on adequate case-mix variable selection, which is time-consuming and costly [110, 111]. A lack of educational support could also play a role, and it may be useful to provide examples of successes and failures with using PROMs data [112]. Finally, aggregated PROMs are used extensively in research aimed at improving quality care through, e.g. identifying subgroups at risk for poorer outcomes. These studies presumably have a large impact on national clinical guidelines, however, to our knowledge, the impact is hardly reported in peer-reviewed literature. The same applies to quality benchmarking under the supervision of professional organisations: this information is discussed with hospital groups and individuals but is generally not published.

Some examples, however, were found for in-depth analysis and PDCA-cycles with the aim to initiate quality improvements. A PDCA-cycle provides a structured and iterative approach to test changes aimed at improving the quality of systems [113]. Four studies were found that exploited these types of methods using PROMs data, all finding a benefit on patient outcomes. Zaslansky et al. suggested that the success could be attributable to the relatively low starting performance of partaking departments [60]. A commonality among these studies is the clear definition of the goal, an action plan, and feedback on the intervention along the way; all potential items which might facilitate the success of a quality improvement initiative, also highlighted by a Cochrane review [114].

### Strengths and limitations

The major strength of this review is the broad search strategy, including the added value of PROMs at the micro-, meso- and macro-level. Several limitations must be acknowledged. Non-peer-reviewed literature (e.g., registry reports), which may be an important source of information on the use of PROMs as quality improvement tool, was excluded. However, this was not deemed feasible because these documents are often published in non-English languages and generally do not report clear evidence of an impact, such as a before-after comparison. Meta-analysis and estimating the effect sizes were not possible due to the heterogeneity of outcomes. PROM scores were variably reported as total score and/or by dimension, limiting the synthesis on the impact of PROMs-interventions by outcome dimensions.

## Conclusion

This systematic review provides a comprehensive overview of novel applications of PROMs which aim improve patient outcomes, and determinants for increased effectiveness. The effectiveness appears to relate to the underlying mechanism, type of PROM used and outcome studied. At the micro-level, for example, PROMs feedback to patient or provider was positively associated with patient outcomes in approximately half of studies. Contemporary studies went a step further and linked PROMs scores to care pathways in for example depression, oncological and gastrointestinal care, which resulted in improved outcomes in a higher percentage of studies. At the meso-/macro-level evidence was limited, and evidence did not suggest a benefit of using PROMs for benchmarking. Promising applications included in-depth analysis and PDCA-cycles using PROMs data. With the increasing use of PROMs in routine clinical care, these findings may help in designing applications which truly impact patient outcomes. As the quality of studies was moderate at best raising concerns regarding the validity of findings, rigorously designed studies should be conducted on testing these applications.

## Supplementary Information

Supplementary Material 1

Supplementary Material 2.

Supplementary Material 3.

## Data Availability

All data generated or analysed during this study are included in this published article.

## Abbreviations

PROMs: Patient-reported outcome measures
PDCA cycle: Plan-do-study-act cycle
PRISMA: Preferred Reporting Items for Systematic Reviews and Meta-Analyses
PREMs: Patient reported experiences measures
HRQoL: Health-Related Quality of Life
ED: Emergency department visits
