# Supplementary material for: The use of patient-reported outcome measures to improve patient-related outcomes – a systematic review
Source: Health Qual Life Outcomes. 2024 Nov 26;22:101. doi: 10.1186/s12955-024-02312-4 (PMC11600902; doi:10.1186/s12955-024-02312-4)
Supplement: Supplementary file 1 — Supplementary Material 1 [file 12955_2024_2312_MOESM1_ESM.docx]

**Additional File 1 Search Strategy**

**Embase.com (1971-)**

('patient-reported outcome'/de OR ('self report'/de AND ('patient satisfaction'/de OR 'treatment outcome'/de OR 'outcome assessment'/de)) OR 'Short Form 36'/exp OR 'European Quality of Life 5 Dimensions questionnaire'/de OR (((patient OR self) NEXT/1 report* NEXT/3 (outcome* OR tool* OR data OR feedback OR satisf*)) OR ((patientreport* OR selfreport* OR Short-Form OR sf) NEXT/3 (outcome* OR tool* OR data OR feedback OR satisf*)) OR ((Veterans-RAND OR VR) NEXT/1 (36 OR 12 OR 6D )) OR PROMIS OR EQ-5D OR prom OR proms):ab,ti) AND ('health care quality'/de OR 'total quality management'/de OR 'quality control'/de OR 'quality control procedures'/de OR 'benchmarking'/de OR 'clinical audit'/de OR (((care OR health-care OR healthcare OR indicator* OR hospital* OR improve* OR control OR doctor* OR personnel* OR assur* OR ensur* OR regist*) NEAR/3 (qualit* OR performance*)) OR benchmark* OR bench-mark* OR audit OR auditing OR audits OR ((care OR health-care OR healthcare OR outcome*) NEAR/3 (improve*)) OR transparency OR (system NEAR/3 inefficien*) OR (practice* NEAR/3 variation*) OR professional­-quality-system):ab,ti) AND ('register'/de OR 'cancer registry'/de OR 'benchmarking'/de OR 'clinical audit'/de OR 'feedback system'/de OR 'decision support system'/exp OR 'data base'/de OR 'minimal important change'/de OR 'plan do study act cycle'/de OR 'funnel plot'/de OR 'monitoring'/de OR (register* OR registr* OR benchmark* OR bench-mark* OR audit OR auditing OR audits OR feedback OR nation-wide OR nationwide OR PDCA OR (Plan-do NEXT/1 (check OR study) NEXT/1 act) OR (minimal* NEAR/3 important NEAR/3 change*) OR cusum OR funnel-plot OR monitoring OR professional­-quality-system OR alert OR stakeholder OR worldwide OR world-wide OR System-Wide OR SystemWide OR national OR international OR (decision NEAR/3 (system* OR tool* OR tree* OR assisted OR support* OR aid OR aids)) OR ((data-base* OR database*) NOT (review*))):ab,ti) NOT ([Conference Abstract]/lim AND [1800-2017]/py) AND [English]/lim

**Medline ALL Ovid (1946-)**

(Patient Reported Outcome Measures/ OR (Self Report/ AND (Patient Satisfaction/ OR Treatment Outcome/ OR Outcome Assessment, Health Care/ OR Patient Outcome Assessment/)) OR (((patient OR self) ADJ report* ADJ3 (outcome* OR tool* OR data OR feedback OR satisf*)) OR ((patientreport* OR selfreport* OR Short-Form OR sf) ADJ3 (outcome* OR tool* OR data OR feedback OR satisf*)) OR ((Veterans-RAND OR VR) ADJ (36 OR 12 OR 6D )) OR PROMIS OR EQ-5D OR prom OR proms).ab,ti.) AND (Quality of Health Care/ OR Total Quality Management/ OR Quality Control/ OR Benchmarking/ OR Clinical Audit/ OR (((care OR health-care OR healthcare OR indicator* OR hospital* OR improve* OR control OR doctor* OR personnel* OR assur* OR ensur* OR regist*) ADJ3 (qualit* OR performance*)) OR benchmark* OR bench-mark* OR audit OR auditing OR audits OR ((care OR health-care OR healthcare OR outcome*) ADJ3 (improve*)) OR transparency OR (system ADJ3 inefficien*) OR (practice* ADJ3 variation*) OR professionall-quality-system).ab,ti.) AND (Registries/ OR Benchmarking/ OR Clinical Audit/ OR feedback system/ OR Decision Support Systems, Management/ OR (register* OR registr* OR benchmark* OR bench-mark* OR audit OR auditing OR audits OR feedback OR nation-wide OR nationwide OR PDCA OR (Plan-do ADJ (check OR study) ADJ act) OR (minimal* ADJ3 important ADJ3 change*) OR cusum OR funnel-plot OR monitoring OR professional-quality-system OR alert OR stakeholder OR worldwide OR world-wide OR System-Wide OR SystemWide OR national OR international OR (decision ADJ3 (system* OR tool* OR tree* OR assisted OR support* OR aid OR aids)) OR ((data-base* OR database*) NOT (review*))).ab,ti.) AND english.la.

**Web of Science Core Collection (1975-)**

AB=(((((patient OR self) NEAR/1 report* NEAR/2 (outcome* OR tool* OR data OR feedback OR satisf*)) OR ((patientreport* OR selfreport* OR Short-Form OR sf) NEAR/2 (outcome* OR tool* OR data OR feedback OR satisf*)) OR ((Veterans-RAND OR VR) NEAR/1 (36 OR 12 OR 6D )) OR PROMIS OR EQ-5D OR prom OR proms)) AND ((((care OR health-care OR healthcare OR indicator* OR hospital* OR improve* OR control OR doctor* OR personnel* OR assur* OR ensur* OR regist*) NEAR/2 (qualit* OR performance*)) OR benchmark* OR bench-mark* OR audit OR auditing OR audits OR ((care OR health-care OR healthcare OR outcome*) NEAR/2 (improve*)) OR transparency OR (system NEAR/2 inefficien*) OR (practice* NEAR/2 variation*) OR professional-quality-system)) AND ((register* OR registr* OR benchmark* OR bench-mark* OR audit OR auditing OR audits OR feedback OR nation-wide OR nationwide OR PDCA OR (Plan-do NEAR/1 (check OR study) NEAR/1 act) OR (minimal* NEAR/2 important NEAR/2 change*) OR cusum OR funnel-plot OR monitoring OR professiona-quality-system OR alert OR stakeholder OR worldwide OR world-wide OR System-Wide OR SystemWide OR national OR international OR (decision NEAR/2 (system* OR tool* OR tree* OR assisted OR support* OR aid OR aids)) OR ((data-base* OR database*) NOT (review*))))) AND DT=(article) AND LA=(english)

**Cochrane CENTRAL register of trials**

((((patient OR self) NEXT/1 report* NEXT/3 (outcome* OR tool* OR data OR feedback OR satisf*)) OR ((patientreport* OR selfreport* OR Short-Form OR sf) NEXT/3 (outcome* OR tool* OR data OR feedback OR satisf*)) OR ((Veterans-RAND OR VR) NEXT/1 (36 OR 12 OR 6D )) OR PROMIS OR EQ-5D OR prom OR proms):ab,ti) AND ((((care OR health-care OR healthcare OR indicator* OR hospital* OR improve* OR control OR doctor* OR personnel* OR assur* OR ensur* OR regist*) NEAR/3 (qualit* OR performance*)) OR benchmark* OR bench-mark* OR audit OR auditing OR audits OR ((care OR health-care OR healthcare OR outcome*) NEAR/3 (improve*)) OR transparency OR (system NEAR/3 inefficien*) OR (practice* NEAR/3 variation*) OR professional-quality-system):ab,ti) AND ((register* OR registr* OR benchmark* OR bench-mark* OR audit OR auditing OR audits OR feedback OR nation-wide OR nationwide OR PDCA OR ("Plan-do" NEXT/1 (check OR study) NEXT/1 "act") OR (minimal* NEAR/3 important NEAR/3 change*) OR cusum OR funnel-plot OR monitoring OR professional-quality-system OR "alert" OR stakeholder OR worldwide OR world-wide OR System-Wide OR SystemWide OR national OR international OR (decision NEAR/3 (system* OR tool* OR tree* OR assisted OR support* OR aid OR aids)) OR ((data-base* OR database*) NOT (review*))):ab,ti)

**Google scholar**

"patient|self reported outcome|tool|data|feedback|satisfaction" "care|healthcare|hospital quality|performance"|"quality|performance indicator"|benchmarking|audit register|registries|benchmarking|audit|nationwide|worldwide|systemWide|national|international
