# Supplementary material for: The use of patient-reported outcome measures to improve patient-related outcomes – a systematic review
Source: Health Qual Life Outcomes. 2024 Nov 26;22:101. doi: 10.1186/s12955-024-02312-4 (PMC11600902; doi:10.1186/s12955-024-02312-4)
Supplement: Supplementary file 2 — Supplementary Material 2. [file 12955_2024_2312_MOESM2_ESM.docx]

**Additional File 2 Description of included studies**

**Micro-level**

Sixty-eight out of 76 studies provided evidence of the use of PROMs at the micro-level (1-70). Two studies were performed in Australia (2, 26), 9 in Canada (6, 7, 25, 28, 31, 49, 53, 57, 62), 1 in Chile (48), 2 in Denmark (39, 61), 1 in Finland (33), 1 in France (27), 1 in Italy (5), 6 in The Netherlands (1, 16-18, 29, 65), 3 in Norway (9, 30, 45), 1 in Sweden (34), 1 in Taiwan (59), 7 in the United Kingdom (UK) (4, 43, 44, 54, 58, 66, 70), 32 in the United States (US) (3, 8, 10-15, 19-24, 35-37, 40, 41, 46, 47, 50-52, 55, 56, 60, 63, 64, 68, 69). One study was performed in multiple countries (42). Studies were conducted in various medical domains: 4 in cardiology (15, 25, 41, 55), 2 in community care (2, 28), 1 in dermatology (4), 5 in gastroenterology (3, 10, 14, 16, 51), 1 in gynecology (45), 1 in midwife care (65), 19 in oncology (7, 8, 13, 18, 22, 26, 29, 31, 33, 39, 42, 46, 47, 50, 54, 56, 58, 61, 66), 6 in orthopedic (trauma) surgery (1, 6, 30, 35, 59, 70) 2 in pediatrics (17, 21), 17 in primary care (5, 12, 19, 20, 23, 24, 36-38, 40, 43, 52, 53, 60, 62, 64, 69), 1 in psychiatry (48), 1 in pulmonary medicine (57), 1 in rehabilitation medicine (9), 4 in rheumatology (11, 27, 63, 68), and 1 in day surgery (34). One study was conducted in a university setting (44). Most studies were performed in the outpatient setting.

Sixty-three studies used a disease-specific instrument in their intervention (1-5, 7, 8, 10, 11, 13-16, 18-22, 24-26, 28, 29, 31-34, 36-39, 41-51, 54-56, 58-61, 63-70), 3 used a generic instrument (6, 40, 57) and 10 a combination of these (7, 9, 12, 23, 27, 30, 35, 52, 53, 62). Fifty-six studies described the mode of PROM administration in the intervention: 8 used pen/paper (2, 23, 36, 37, 44, 52, 56, 57, 65), 5 interviewed patients (12, 28, 43, 53, 59), 8 contacted patients via telephone (25, 38, 41, 46, 47, 55, 58, 70), 2 used an automated telephone interview system (26, 69), 29 used an e-portal (3, 4, 6-11, 13, 14, 16, 17, 27, 29-31, 33, 34, 39, 40, 42, 45, 48, 49, 54, 60, 62, 63, 66), and 4 a combination (20, 50, 64). Twenty-eight studies represented a form of training to the providers of the intervention: 16 provided training on the interpretation of the PROMs and/or use of the tool (1, 10, 11, 17, 18, 23, 28, 29, 35, 42, 54, 57, 61-63, 66), while 3 also explicitly mentioned training on the use of guidelines (31, 65, 69). Nine studies mentioned training on co-interventions and/or study design (2, 20, 21, 26, 37, 38, 43, 46, 64).

**Meso-/macro-level**

Eight out of 76 studies provided evidence on the use of PROMs at the meso-/macro-level (71-78). Four studies were rated as moderate quality (72-74, 76), while the other 4 were rated as weak quality (71, 75, 77, 78).

One study was conducted in Canada (73), 1 in Ireland (71), 1 in Mexico (78), 1 in Switzerland (72), 2 in the UK (74, 76), 1 in the US (77), and 1 in multiple countries (75). Three studies included multiple non-surgical and surgical specialties (72, 74, 75), 1 in only surgical specialties (78), 2 in orthopedic surgery (71, 76), 1 in urological surgery (73), and 1 in primary care (77). Two studies were conducted nationwide (71, 74), 4 at inpatient departments (72, 73, 75, 78), 1 in outpatients (77), and 1 at an unspecified (i.e., inpatient or outpatient) department (76). Five studies used a disease-specific PROM (71-73, 75, 78) in their intervention, 1 used a generic PROM (77), and 2 used both a disease-specific and generic PROM (74, 76).

The method of PROMs collection was only explicitly described in 1 study, which used mail and telephone surveys (77). Five studies described training of the providers in the employed intervention: 2 described training on the study design only (75, 78), 1 on the use of guidelines relevant to the patient population (72), 1 on the interpretation of PROMs (71), and 1 on the new surgical technique introduced (76).

**REFERENCES**

1. Ackermans L, Hageman MG, Bos AH, Haverkamp D, Scholtes VAB, Poolman RW. Feedback to Patients About Patient-reported Outcomes Does Not Improve Empowerment or Satisfaction. Clin Orthop Relat Res. 2018;476(4):716-22.

2. Allen J, Annells M, Nunn R, Petrie E, Clark E, Lang L, et al. Evaluation of effectiveness and satisfaction outcomes of a mental health screening and referral clinical pathway for community nursing care. J Psychiatr Ment Health Nurs. 2011;18(5):375-85.

3. Almario CV, Chey WD, Khanna D, Mosadeghi S, Ahmed S, Afghani E, et al. Impact of National Institutes of Health Gastrointestinal PROMIS Measures in Clinical Practice: Results of a Multicenter Controlled Trial. Am J Gastroenterol. 2016;111(11):1546-56.

4. Baker A, Mitchell EJ, Partlett C, Thomas KS. Evaluating the effect of weekly patient-reported symptom monitoring on trial outcomes: results of the Eczema Monitoring Online randomized controlled trial. Br J Dermatol. 2023;189(2):180-7.

5. Balestrieri M, Sisti D, Rocchi M, Rucci P, Simon G, Araya R, et al. Effectiveness of clinical decision support systems and telemedicine on outcomes of depression: a cluster randomized trial in general practice. Fam Pract. 2020;37(6):731-7.

6. Bansback N, Trenaman L, MacDonald KV, Durand D, Hawker G, Johnson JA, et al. An online individualised patient decision aid improves the quality of decisions in patients considering total knee arthroplasty in routine care: A randomized controlled trial. Osteoarthr Cartil Open. 2022;4(3):100286.

7. Barbera L, Sutradhar R, Howell D, Sussman J, Seow H, Dudgeon D, et al. Does routine symptom screening with ESAS decrease ED visits in breast cancer patients undergoing adjuvant chemotherapy? Support Care Cancer. 2015;23(10):3025-32.

8. Basch E, Deal AM, Kris MG, Scher HI, Hudis CA, Sabbatini P, et al. Symptom Monitoring With Patient-Reported Outcomes During Routine Cancer Treatment: A Randomized Controlled Trial. J Clin Oncol. 2016;34(6):557-65.

9. Berdal G, Sand-Svartrud AL, Linge AD, Aasvold AM, Tennebø K, Eppeland SG, et al. Bridging gaps across levels of care in rehabilitation of patients with rheumatic and musculoskeletal diseases: Results from a stepped-wedge cluster randomized controlled trial. Clin Rehabil. 2023;37(9):1153-77.

10. Berinstein JA, Cohen-Mekelburg SA, Greenberg GM, Wray D, Berry SK, Saini SD, et al. A Care Coordination Intervention Improves Symptoms But Not Charges in High-Risk Patients With Inflammatory Bowel Disease. Clin Gastroenterol Hepatol. 2022;20(5):1029-38 e9.

11. Buckley L, Ware E, Kreher G, Wiater L, Mehta J, Burnham JM. Outcome Monitoring and Clinical Decision Support in Polyarticular Juvenile Idiopathic Arthritis. J Rheumatol. 2020;47(2):273-81.

12. Buxton JA, Chandler-Altendorf A, Puente AE. A novel collaborative practice model for treatment of mental illness in indigent and uninsured patients. Am J Health Syst Pharm. 2012;69(12):1054-62.

13. Cooley ME, Mazzola E, Xiong N, Hong F, Lobach DF, Braun IM, et al. Clinical Decision Support for Symptom Management in Lung Cancer Patients: A Group RCT. J Pain Symptom Manage. 2022;63(4):572-80.

14. Cross RK, Langenberg P, Regueiro M, Schwartz DA, Tracy JK, Collins JF, et al. A Randomized Controlled Trial of TELEmedicine for Patients with Inflammatory Bowel Disease (TELE-IBD). Am J Gastroenterol. 2019;114(3):472-82.

15. Davidson KW, Rieckmann N, Clemow L, Schwartz JE, Shimbo D, Medina V, et al. Enhanced depression care for patients with acute coronary syndrome and persistent depressive symptoms: coronary psychosocial evaluation studies randomized controlled trial. Arch Intern Med. 2010;170(7):600-8.

16. de Jong MJ, van der Meulen-de Jong AE, Romberg-Camps MJ, Becx MC, Maljaars JP, Cilissen M, et al. Telemedicine for management of inflammatory bowel disease (myIBDcoach): a pragmatic, multicentre, randomised controlled trial. Lancet. 2017;390(10098):959-68.

17. de Wit M, Delemarre-van de Waal HA, Bokma JA, Haasnoot K, Houdijk MC, Gemke RJ, et al. Monitoring and discussing health-related quality of life in adolescents with type 1 diabetes improve psychosocial well-being: a randomized controlled trial. Diabetes Care. 2008;31(8):1521-6.

18. Detmar SB, Muller MJ, Schornagel JH, Wever LD, Aaronson NK. Health-related quality-of-life assessments and patient-physician communication: a randomized controlled trial. Jama. 2002;288(23):3027-34.

19. Dhingra L, Schiller R, Teets R, Nosal S, Dieckmann NF, Ginzburg R, et al. Pain Management in Primary Care: A Randomized Controlled Trial of a Computerized Decision Support Tool. Am J Med. 2021;134(12):1546-54.

20. Dobscha SK, Corson K, Hickam DH, Perrin NA, Kraemer DF, Gerrity MS. Depression decision support in primary care: a cluster randomized trial. Ann Intern Med. 2006;145(7):477-87.

21. Epstein JN, Rabiner D, Johnson DE, Fitzgerald DP, Chrisman A, Erkanli A, et al. Improving attention-deficit/hyperactivity disorder treatment outcomes through use of a collaborative consultation treatment service by community-based pediatricians: a cluster randomized trial. Arch Pediatr Adolesc Med. 2007;161(9):835-40.

22. Ferrell B, Chung V, Hughes MT, Koczywas M, Azad NS, Ruel NH, et al. A Palliative Care Intervention for Patients on Phase 1 Studies. J Palliat Med. 2021;24(6):846-56.

23. Fihn SD, McDonell MB, Diehr P, Anderson SM, Bradley KA, Au DH, et al. Effects of sustained audit/feedback on self-reported health status of primary care patients. Am J Med. 2004;116(4):241-8.

24. Fortmann AL, Walker C, Barger K, Robacker M, Morrisey R, Ortwine K, et al. Care Team Integration in Primary Care Improves One-Year Clinical and Financial Outcomes in Diabetes: A Case for Value-Based Care. Popul Health Manag. 2020;23(6):467-75.

25. Frasure-Smith N, Lespérance F, Prince RH, Verrier P, Garber RA, Juneau M, et al. Randomised trial of home-based psychosocial nursing intervention for patients recovering from myocardial infarction. Lancet. 1997;350(9076):473-9.

26. Girgis A, Breen S, Stacey F, Lecathelinais C. Impact of two supportive care interventions on anxiety, depression, quality of life, and unmet needs in patients with nonlocalized breast and colorectal cancers. J Clin Oncol. 2009;27(36):6180-90.

27. Gossec L, Cantagrel A, Soubrier M, Berthelot JM, Joubert JM, Combe B, et al. An e-health interactive self-assessment website (Sanoia(®)) in rheumatoid arthritis. A 12-month randomized controlled trial in 320 patients. Joint Bone Spine. 2018;85(6):709-14.

28. Hadjistavropoulos T, MacNab Y, Lints-Martindale A, Martin R, Hadjistavropoulos H. Does routine pain assessment result in better care? Pain Res Manag. 2009;14(3):211-6.

29. Hilarius DL, Kloeg PH, Gundy CM, Aaronson NK. Use of health-related quality-of-life assessments in daily clinical oncology nursing practice: a community hospital-based intervention study. Cancer. 2008;113(3):628-37.

30. Holm I, Pripp AH, Risberg MA. The Active with OsteoArthritis (AktivA) Physiotherapy Implementation Model: A Patient Education, Supervised Exercise and Self-Management Program for Patients with Mild to Moderate Osteoarthritis of the Knee or Hip Joint. A National Register Study with a Two-Year Follow-Up. J Clin Med. 2020;9(10).

31. Howell D, Li M, Sutradhar R, Gu S, Iqbal J, O'Brien MA, et al. Integration of patient-reported outcomes (PROs) for personalized symptom management in "real-world" oncology practices: a population-based cohort comparison study of impact on healthcare utilization. Support Care Cancer. 2020;28(10):4933-42.

32. Howell D, Rosberger Z, Mayer C, Faria R, Hamel M, Snider A, et al. Personalized symptom management: a quality improvement collaborative for implementation of patient reported outcomes (PROs) in 'real-world' oncology multisite practices. Journal of patient-reported outcomes. 2020;4(1):47.

33. Livanainen S, Ravichandra R, Jekunen A, Arokoski R, Mentu S, Lang L, et al. ePRO symptom follow-up of colorectal cancer patients receiving oxaliplatin-based adjuvant chemotherapy is feasible and enhances the quality of patient care: a prospective multicenter study. J Cancer Res Clin Oncol. 2023;149(10):6875-82.

34. Jaensson M, Dahlberg K, Eriksson M, Nilsson U. Evaluation of postoperative recovery in day surgery patients using a mobile phone application: a multicentre randomized trial. Br J Anaesth. 2017;119(5):1030-8.

35. Jayakumar P, Moore MG, Furlough KA, Uhler LM, Andrawis JP, Koenig KM, et al. Comparison of an Artificial Intelligence-Enabled Patient Decision Aid vs Educational Material on Decision Quality, Shared Decision-Making, Patient Experience, and Functional Outcomes in Adults With Knee Osteoarthritis: A Randomized Clinical Trial. JAMA Netw Open. 2021;4(2):e2037107.

36. Katon W, Robinson P, Von Korff M, Lin E, Bush T, Ludman E, et al. A multifaceted intervention to improve treatment of depression in primary care. Arch Gen Psychiatry. 1996;53(10):924-32.

37. Katon WJ, Von Korff M, Lin EH, Simon G, Ludman E, Russo J, et al. The Pathways Study: a randomized trial of collaborative care in patients with diabetes and depression. Arch Gen Psychiatry. 2004;61(10):1042-9.

38. Katzelnick DJ, Simon GE, Pearson SD, Manning WG, Helstad CP, Henk HJ, et al. Randomized trial of a depression management program in high utilizers of medical care. Arch Fam Med. 2000;9(4):345-51.

39. Kjaer T, Dalton SO, Andersen E, Karlsen R, Nielsen AL, Hansen MK, et al. A controlled study of use of patient-reported outcomes to improve assessment of late effects after treatment for head-and-neck cancer. Radiother Oncol. 2016;119(2):221-8.

40. Kroenke K, Talib TL, Stump TE, Kean J, Haggstrom DA, DeChant P, et al. Incorporating PROMIS Symptom Measures into Primary Care Practice-a Randomized Clinical Trial. J Gen Intern Med. 2018;33(8):1245-52.

41. Kronish IM, Moise N, Cheung YK, Clarke GN, Dolor RJ, Duer-Hefele J, et al. Effect of Depression Screening After Acute Coronary Syndromes on Quality of Life: The CODIACS-QoL Randomized Clinical Trial. JAMA Intern Med. 2020;180(1):45-53.

42. Maguire R, McCann L, Kotronoulas G, Kearney N, Ream E, Armes J, et al. Real time remote symptom monitoring during chemotherapy for cancer: European multicentre randomised controlled trial (eSMART). Bmj. 2021;374:n1647.

43. Mallen CD, Nicholl BI, Lewis M, Bartlam B, Green D, Jowett S, et al. The effects of implementing a point-of-care electronic template to prompt routine anxiety and depression screening in patients consulting for osteoarthritis (the Primary Care Osteoarthritis Trial): A cluster randomised trial in primary care. PLoS Med. 2017;14(4):e1002273.

44. McCambridge J, Day M. Randomized controlled trial of the effects of completing the Alcohol Use Disorders Identification Test questionnaire on self-reported hazardous drinking. Addiction. 2008;103(2):241-8.

45. Ngo E, Truong MB, Wright D, Nordeng H. Impact of a Mobile Application for Tracking Nausea and Vomiting During Pregnancy (NVP) on NVP Symptoms, Quality of Life, and Decisional Conflict Regarding NVP Treatments: MinSafeStart Randomized Controlled Trial. JMIR Mhealth Uhealth. 2022;10(7):e36226.

46. Patel MI, Kapphahn K, Dewland M, Aguilar V, Sanchez B, Sisay E, et al. Effect of a Community Health Worker Intervention on Acute Care Use, Advance Care Planning, and Patient-Reported Outcomes Among Adults With Advanced Stages of Cancer: A Randomized Clinical Trial. JAMA Oncol. 2022;8(8):1139-48.

47. Patel MI, Ramirez D, Agajanian R, Agajanian H, Bhattacharya J, Bundorf KM. Lay Health Worker-Led Cancer Symptom Screening Intervention and the Effect on Patient-Reported Satisfaction, Health Status, Health Care Use, and Total Costs: Results From a Tri-Part Collaboration. JCO Oncol Pract. 2020;16(1):e19-e28.

48. Pérez JC, Fernández O, Cáceres C, Carrasco Á E, Moessner M, Bauer S, et al. An Adjunctive Internet-Based Intervention to Enhance Treatment for Depression in Adults: Randomized Controlled Trial. JMIR Ment Health. 2021;8(12):e26814.

49. Pooni A, Brar MS, Anpalagan T, Schmocker S, Rashid S, Goldstein R, et al. Home to Stay: A Randomized Controlled Trial Evaluating the Effect of a Postdischarge Mobile App to Reduce 30-Day Readmission Following Elective Colorectal Surgery. Ann Surg. 2023;277(5):e1056-e62.

50. Price S, Hamann HA, Halaby L, Trejo J, Rogers FC, Weihs K. Collaborative depression care sensitive to the needs of underserved patients with cancer: Feasibility, acceptability and outcomes. J Psychosoc Oncol. 2023:1-23.

51. Regueiro M, Click B, Anderson A, Shrank W, Kogan J, McAnallen S, et al. Reduced Unplanned Care and Disease Activity and Increased Quality of Life After Patient Enrollment in an Inflammatory Bowel Disease Medical Home. Clin Gastroenterol Hepatol. 2018;16(11):1777-85.

52. Reiber GE, Au D, McDonell M, Fihn SD. Diabetes quality improvement in Department of Veterans Affairs Ambulatory Care Clinics: a group-randomized clinical trial. Diabetes Care. 2004;27 Suppl 2:B61-8.

53. Richardson J, Chan D, Risdon K, Giles C, Mulveney S, Cripps D. Does monitoring change in function in community-dwelling older adults alter outcome? A randomized controlled trial. Clin Rehabil. 2008;22(12):1061-70.

54. Rogers SN, Allmark C, Bekiroglu F, Edwards RT, Fabbroni G, Flavel R, et al. Improving quality of life through the routine use of the patient concerns inventory for head and neck cancer patients: main results of a cluster preference randomised controlled trial. Eur Arch Otorhinolaryngol. 2021;278(9):3435-49.

55. Rollman BL, Anderson AM, Rothenberger SD, Abebe KZ, Ramani R, Muldoon MF, et al. Efficacy of Blended Collaborative Care for Patients With Heart Failure and Comorbid Depression: A Randomized Clinical Trial. JAMA Intern Med. 2021;181(10):1369-80.

56. Rosenbloom SK, Victorson DE, Hahn EA, Peterman AH, Cella D. Assessment is not enough: a randomized controlled trial of the effects of HRQL assessment on quality of life and satisfaction in oncology clinical practice. Psycho-oncology. 2007;16(12):1069-79.

57. Santana MJ, Feeny D, Johnson JA, McAlister FA, Kim D, Weinkauf J, et al. Assessing the use of health-related quality of life measures in the routine clinical care of lung-transplant patients. Quality of life research : an international journal of quality of life aspects of treatment, care and rehabilitation. 2010;19(3):371-9.

58. Sharpe M, Walker J, Holm Hansen C, Martin P, Symeonides S, Gourley C, et al. Integrated collaborative care for comorbid major depression in patients with cancer (SMaRT Oncology-2): a multicentre randomised controlled effectiveness trial. Lancet. 2014;384(9948):1099-108.

59. Shyu YI, Liang J, Tseng MY, Li HJ, Wu CC, Cheng HS, et al. Comprehensive and subacute care interventions improve health-related quality of life for older patients after surgery for hip fracture: a randomised controlled trial. Int J Nurs Stud. 2013;50(8):1013-24.

60. Simon GE, Ralston JD, Savarino J, Pabiniak C, Wentzel C, Operskalski BH. Randomized trial of depression follow-up care by online messaging. J Gen Intern Med. 2011;26(7):698-704.

61. Skovlund PC, Vind Thaysen H, Schmidt H, Alsner J, Hjollund NH, Lomborg K, et al. Effect of patient-reported outcomes as a dialogue-based tool in cancer consultations on patient self-management and health-related quality of life: a clinical, controlled trial. Acta Oncol. 2021;60(12):1668-77.

62. Steele Gray C, Chau E, Tahsin F, Harvey S, Loganathan M, McKinstry B, et al. Assessing the Implementation and Effectiveness of the Electronic Patient-Reported Outcome Tool for Older Adults With Complex Care Needs: Mixed Methods Study. J Med Internet Res. 2021;23(12):e29071.

63. Tirelli F, Xiao R, Brandon TG, Burnham JM, Chang JC, Weiss PF. Determinants of disease activity change over time in Enthesitis related arthritis: effect of structured outcome monitoring and clinical decision support. Pediatr Rheumatol Online J. 2020;18(1):79.

64. Unützer J, Katon W, Callahan CM, Williams JW, Jr., Hunkeler E, Harpole L, et al. Collaborative care management of late-life depression in the primary care setting: a randomized controlled trial. Jama. 2002;288(22):2836-45.

65. van der Zee-van den Berg AI, Boere-Boonekamp MM, Groothuis-Oudshoorn CGM, MJ IJ, Haasnoot-Smallegange RME, Reijneveld SA. Post-Up Study: Postpartum Depression Screening in Well-Child Care and Maternal Outcomes. Pediatrics. 2017;140(4).

66. Velikova G, Booth L, Smith AB, Brown PM, Lynch P, Brown JM, et al. Measuring quality of life in routine oncology practice improves communication and patient well-being: a randomized controlled trial. J Clin Oncol. 2004;22(4):714-24.

67. Velikova G, Keding A, Harley C, Cocks K, Booth L, Smith AB, et al. Patients report improvements in continuity of care when quality of life assessments are used routinely in oncology practice: secondary outcomes of a randomised controlled trial. Eur J Cancer. 2010;46(13):2381-8.

68. Volkmann ER, FitzGerald JD. Reducing gender disparities in post-total knee arthroplasty expectations through a decision aid. BMC Musculoskelet Disord. 2015;16(1):16.

69. Wu S, Ell K, Jin H, Vidyanti I, Chou CP, Lee PJ, et al. Comparative Effectiveness of a Technology-Facilitated Depression Care Management Model in Safety-Net Primary Care Patients With Type 2 Diabetes: 6-Month Outcomes of a Large Clinical Trial. J Med Internet Res. 2018;20(4):e147.

70. Wylde V, Bertram W, Sanderson E, Noble S, Howells N, Peters TJ, et al. The STAR care pathway for patients with pain at 3 months after total knee replacement: a multicentre, pragmatic, randomised, controlled trial. Lancet Rheumatol. 2022;4(3):e188-e97.

71. Boyce MB, Browne JP. The effectiveness of providing peer benchmarked feedback to hip replacement surgeons based on patient-reported outcome measures--results from the PROFILE (Patient-Reported Outcomes: Feedback Interpretation and Learning Experiment) trial: a cluster randomised controlled study. BMJ open. 2015;5(7):e008325.

72. Haller G, Agoritsas T, Luthy C, Piguet V, Griesser AC, Perneger T. Collaborative quality improvement to manage pain in acute care hospitals. Pain Med. 2011;12(1):138-47.

73. Kumar RM, Fergusson DA, Lavallée LT, Cagiannos I, Morash C, Horrigan M, et al. Performance Feedback May Not Improve Radical Prostatectomy Outcomes: The Surgical Report Card (SuRep) Study. J Urol. 2021;206(2):346-53.

74. Varagunam M, Hutchings A, Neuburger J, Black N. Impact on hospital performance of introducing routine patient reported outcome measures in surgery. Journal of health services research & policy. 2014;19(2):77-84.

75. Zaslansky R, Chapman CR, Baumbach P, Bytyqi A, Castro Lopes JM, Chetty S, et al. Improving perioperative pain management: a preintervention and postintervention study in 7 developing countries. Pain Rep. 2019;4(1):e705.

76. Partridge T, Carluke I, Emmerson K, Partington P, Reed M. Improving patient reported outcome measures (PROMs) in total knee replacement by changing implant and preserving the infrapatella fatpad: a quality improvement project. BMJ quality improvement reports. 2016;5(1).

77. Weingarten SR, Kim CS, Stone EG, Kristopaitis RJ, Pelter M, Sandhu M. Can peer-comparison feedback improve patient functional status? The American journal of managed care. 2000;6(1):35-9.

78. Garduño-López AL, Acosta Nava VM, Castro Garcés L, Rascón-Martínez DM, Cuellar-Guzmán LF, Flores-Villanueva ME, et al. Towards Better Perioperative Pain Management in Mexico: A Study in a Network of Hospitals Using Quality Improvement Methods from PAIN OUT. J Pain Res. 2021;14:415-30.
